# Continuous renal replacement therapy in neonates and children: what does the pediatrician need to know? An overview from the Critical Care Nephrology Section of the European Society of Paediatric and Neonatal Intensive Care (ESPNIC)

**DOI:** 10.1007/s00431-023-05318-0

**Published:** 2023-11-17

**Authors:** Gerard Cortina, Marco Daverio, Demet Demirkol, Rahul Chanchlani, Akash Deep

**Affiliations:** 1 Department of Pediatrics, Medical University of Innsbruck, Innsbruck, Austria; 2 Pediatric Intensive Care Unit, University Hospital of Padua, Padua, Italy; 3 Pediatric Intensive Care Unit, Faculty of Medicine, Istanbul University, Istanbul, Turkey; 4 Division of Pediatric Nephrology, Department of Pediatrics, McMaster Children’s Hospital, McMaster University, Hamilton, ON Canada; 5 Pediatric Intensive Care Unit, Kings College London, London, UK

**Keywords:** Critically ill children, Continuous renal replacement therapy, Acute kidney injury, Fluid overload, Follow-up

## Abstract

Continuous renal replacement therapy (CRRT) is the preferred method for renal support in critically ill and hemodynamically unstable children in the pediatric intensive care unit (PICU) as it allows for gentle removal of fluids and solutes. The most frequent indications for CRRT include acute kidney injury (AKI) and fluid overload (FO) as well as non-renal indications such as removal of toxic metabolites in acute liver failure, inborn errors of metabolism, and intoxications and removal of inflammatory mediators in sepsis. AKI and/or FO are common in critically ill children and their presence is associated with worse outcomes. Therefore, early recognition of AKI and FO is important and timely transfer of patients who might require CRRT to a center with institutional expertise should be considered. Although CRRT has been increasingly used in the critical care setting, due to the lack of standardized recommendations, wide practice variations exist regarding the main aspects of CRRT application in critically ill children.

*Conclusion*: In this review, from the Critical Care Nephrology section of the European Society of Paediatric and Neonatal Intensive Care (ESPNIC), we summarize the key aspects of CRRT delivery and highlight the importance of adequate follow up among AKI survivors which might be of relevance for the general pediatric community.

**Table:** 

**What is Known:** *• CRRT is the preferred method of renal support in critically ill and hemodynamically unstable children in the PICU as it allows for gentle removal of fluids and solutes*. *• Although CRRT has become an important and integral part of modern pediatric critical care, wide practice variations exist in all aspects of CRRT*.	
**What is New:** *• Given the lack of literature on guidance for a general pediatrician on when to refer a child for CRRT, we recommend timely transfer to a center with institutional expertise in CRRT, as both worsening AKI and FO have been associated with increased mortality*. *• Adequate follow-up of PICU patients with AKI and CRRT is highlighted as recent findings demonstrate that these children are at increased risk for adverse long-term outcomes*.

**What is Known:**

*• CRRT is the preferred method of renal support in critically ill and hemodynamically unstable children in the PICU as it allows for gentle removal of fluids and solutes*.

*• Although CRRT has become an important and integral part of modern pediatric critical care, wide practice variations exist in all aspects of CRRT*.

**What is New:**

*• Given the lack of literature on guidance for a general pediatrician on when to refer a child for CRRT, we recommend timely transfer to a center with institutional expertise in CRRT, as both worsening AKI and FO have been associated with increased mortality*.

*• Adequate follow-up of PICU patients with AKI and CRRT is highlighted as recent findings demonstrate that these children are at increased risk for adverse long-term outcomes*.

## Introduction

Continuous renal replacement therapy (CRRT) is the preferred method of renal support in critically ill children in the pediatric intensive care unit (PICU) as it allows for continuous and controlled fluid and solute clearance in hemodynamically unstable patients [1–6]. In contrast, intermittent modalities like hemodialysis are applied in stable patients outside of ICU and in the outpatient setting [7–9]. The use of CRRT in the PICU has been rising due to standardized definitions, and therefore earlier recognition of acute kidney injury (AKI) as well as fluid overload (FO); moreover, in recent years, a growing number of patients with sepsis, following cardiac surgery or respiratory failure [10–14], are at increased risk of AKI. Thus, CRRT has become an important and integral part of modern pediatric critical care. Moreover, in the last decades, technical refinements have made CRRT safer, even in small children, and new dedicated machines for the use in small children have found their way in daily practice [15, 16].

However, a recent survey of the Critical Care Nephrology section of the European Society of Paediatric and Neonatal Intensive Care (ESPNIC) demonstrated that wide practice variations exist in all aspects of CRRT, from timing of initiation, vascular access, modality and dose delivery, anticoagulation method, discontinuation of CRRT, among others, as well as follow-up of PICU survivors [17].

The purpose of this review is to give an overview on the indications and key technical aspects, and discuss major controversies as wells as future research aspects in the management of CRRT.

### Indications for CRRT

The most common indications for CRRT in critically ill children are AKI and FO. AKI is common and occurs in 10% of hospitalized children and 30–60% among critically ill children and is associated with adverse short- and long-term outcomes [13, 18–23]. Due to introduction of a standardized definition by KDIGO (Kidney Disease: improving global outcomes) and pRIFLE (Pediatric Risk, Injury, Failure, Loss, End Stage Renal Disease) criteria, AKI is more commonly and earlier diagnosed [10, 11]. The AWARE study recently evaluated the incidence of AKI among critically ill children demonstrating that one of four children admitted to 32 PICUs worldwide developed AKI and 12.6% developed severe AKI, defined as stage KDIGO 2 and 3. Moreover, this study highlighted that mortality rises with increasing severity of AKI (11% in severe AKI vs. 2.6% in AKI stage 1 or no AKI) and the need for CRRT [19].

Besides AKI, FO is common in critically ill children [24–29]. FO is most commonly defined according to the formula described by Goldstein et al.: % FO = Fluid in − fluid out / intensive care admission body weight in kg × 100 [30]. Although FO might occur without AKI, more often, patients at risk of severe FO often are the same who are at risk for AKI and include patients with sepsis, those with cardiac surgery and respiratory failure. The association between the severity of FO and increased mortality has been demonstrated in several studies including a recent meta-analysis which showed a 6% increase in the odds of mortality for every 1% increase in FO [31]. However, in clinical practice, fluid status is often difficult to assess and no absolute surrogate marker for FO exists. Therefore, fluid stewardship and fluid restriction in critically ill children after the initial resuscitation phase are very important to prevent severe FO.

Acid–base and severe electrolyte abnormalities are often associated with AKI [32, 33]. Severe metabolic acidosis unresponsive to conventional medical therapy might trigger earlier initiation of CRRT, especially in patients with acute respiratory distress syndrome (ARDS) and lung-protective ventilation as the combination of respiratory and metabolic acidosis may result in severe acidemia. Other electrolyte abnormalities such as hyperkalemia, hyponatremia, and hyperphosphatemia may accompany AKI and should be considered in the decision-making to initiate CRRT. Severe hyperkalemia may occur without AKI, for example, in the case of tumor lysis syndrome and initiation of CRRT is recommended when potassium levels raise > 6.5 mmol/L despite medical therapy [33].

In addition, there are a variety of non-renal indications for CRRT. Elimination of toxins in patients with inborn errors of metabolism is well established, although CRRT is mainly indicated for ammonia removal as well as in acute liver failure patients, where the early initiation of CRRT to eliminate ammonia and other water soluble toxins has been associated with improved survival as bridge to recovery or bridge to transplant strategy [34–36]. The elimination of cytokines and inflammatory mediators in sepsis-induced multi-organ dysfunction syndrome (MODS) as an immunomodulatory approach has received increasing attention in recent years and some adult studies have demonstrated positive effects on survival [13, 37, 38]. For these purposes, combination of CRRT with other extracorporeal techniques has been used, like therapeutic plasma exchange (TPE) or the addition of specific filters like Cytosorb^®^ or oXiris^®^ which add adsorption to the other CRRT mechanisms [39–41]. Table 1 summarizes the most common indications for CRRT in critically ill children.

**Table 1:** Most common indications for continuous renal replacement therapy initiation in critically ill children

Acute kidney injury with oligo/anuria (< 0.5 ml/kg/h)
Fluid overload > 10%
Severe electrolyte imbalance refractory to medical treatment
Metabolic abnormalities (e.g., hyperammonemia refractory to medical treatment)
Severe metabolic acidosis
Uremic complications(e.g., encephalopathy, pericardial effusion, pulmonary edema)
Intoxications (e.g., drugs and toxins)
Septic shock with need of toxins clearance (e.g., endotoxins, cytokines)
Need to make room for more fluids for drug therapy and/or nutrition

### Timing of initiation

Despite the increased use of CRRT, identifying the optimal timing of initiation remains a difficult decision in clinical practice. On the one hand, an early initiation strategy might result in improved outcomes, especially in patients with conditions with high risk for AKI and significant FO; several retrospective pediatric studies have shown an association between increased mortality and higher degree of fluid overload at CRRT initiation [25, 29, 30, 42]. On the other hand, CRRT is an invasive therapy and may be associated with complications especially in smaller children. Therefore, a more conservative “wait and watch” strategy may avoid overtreatment as some patients might recover and without needing any CRRT treatment. Modem et al. showed a significant difference towards earlier initiation of CRRT in pediatric survivors when compared with non-survivors (2 vs. 3.4 days) [42]. Additionally, the study by Cortina et al. showed that the odds of mortality increased by 1% for every hour of delay in CRRT initiation [29]. However, these were single-center retrospective studies and therefore results may not be generalizable. Optimal timing of initiation of CRRT has been studied in several RCT’s in adult patients with mixed results [43, 44]. The most recent multicenter AKIKI2 trial showed that a more delayed CRRT strategy led to a reduction in CRRT use. However, on the other hand, the hazard for death at 60 days increased significantly in the more delayed strategy group [45]. Taken together, given current available evidence, most experts would advise to consider initiation of CRRT in critically ill children with FO > 10% when diuretics are unable to reverse or maintain fluid balance [46].

## Key aspects of CRRT delivery

### Vascular access

The performance and delivery of CRRT depends heavily on an efficient vascular access [47, 48]. Vascular access is essential in achieving adequate blood flow rates, which prolongs circuit lifetime (CL) and thus reduces interruptions while optimizing the delivered CRRT dose. However, vascular access may be challenging especially in newborns and infants and the availability of adequate dialysis catheters, especially for small children, remains problematic. In order to minimize complications, KDIGO recommends placement of an adequate central line using ultrasound guidance [11]. The most important factor ensuring low resistance during high blood flow rates is the location of the catheter tip and its diameter. The catheter should be long enough so that the tip resides at the superior cavoatrial junction when using the upper body approach, or the inferior vena cava when using the femoral approach. The right internal jugular vein is recommended as the first choice because of its straight course into the right atrium, which leads to less contact with the vessel wall, and thus better flow and lower risk of catheter-associated central vein thrombosis [48]. The femoral vein is considered second choice and a good option in pediatrics, as it is easily accessible in children. KDIGO recommends that the subclavian vein should be avoided due to the increased risk of insertion complications and of central vein thrombosis. However, recent literature suggests that cannulation of the left brachiocephalic vein, using the supra- or infraclavicular ultrasound-guided approach, is an excellent choice in neonates and small infants, due to the large caliber of this vessel and due to the fact that it is non-collapsible [49–52]. Although renal function will recover in the majority of children with AKI, the long-term vascular health of a patient requiring CRRT should always be considered, and in order to prevent thromboembolic complications, a catheter-to-vessel ratio of 45% should not be exceeded [53].

### CRRT modality and dose

Continuous modalities are most frequently used in critically ill children as it allows for gentle fluid removal and minimizes fluid shifts and therefore is preferred in critically ill children at risk of severe hypotension or cerebral edema [8, 9, 54]. In contrast, intermittent renal replacement therapies like hemodialysis (iHD) are frequently used in stable patients with AKI outside the ICU and in the outpatient setting in chronic kidney disease (CKD). iHD may be useful or preferable in a few clinical scenarios when rapid elimination of small molecules like electrolytes or toxins is required such as in case of life-threatening hyperkalemia, as it allows for rapid solute clearance and ultrafiltration during relatively short treatment sessions [32, 33]. Peritoneal dialysis (PD) is another alternative to CRRT, according to local preferences and expertise [55, 56]. The advantage of PD is that it is possible in newborns, easy to perform without requiring complex technology, and is cheaper than CRRT. However, it is contra-indicated in children with abdominal pathology or surgery, requires a surgical intervention for the catheter insertion, and results in lower clearance and ultrafiltration volumes compared to extracorporeal therapies, as fluid and/or solute removal rates are dependent on the diffusion capacity of the peritoneum. Recently, continuous flow peritoneal dialysis (CFPD) has been proposed as effective technique to improve ultrafiltration in children with AKI and FO [57]. Table 2 summarizes the pro and cons of the different CRRT modalities, as well as its alternatives iHD and PD and Table 3 illustrates the main characteristics of PD compared to extracorporeal therapies.

**Table 2:** Advantages and disadvantages of the different CRRT modalities, as well as its alternatives iHD and PD

**Technique**	**Physical principle**	**Minimum duration (hours)**	**Advantages**	**Disadvantages**
**PD**	Diffusion	24	• Technically, the simplest modality • Require less infrastructure and lower costs • No need for anticoagulation • Possible in hemodynamically unstable patients	• Slow small molecule and uremic toxin clearance • Less predictable fluid removal • Risk of infections (e.g., peritonitis) • Not possible if recent abdominal surgery • May have impact on respiratory stability
**IHD**	Diffusion	4–6	• Rapid removal of toxins, electrolytes and fluid overload • Minimal/no need for anticoagulation • Relatively lower cost than the techniques below • Less restrictions on patients mobility	• Not recommended in critically ill hemodynamically unstable patients • Increased risk of hypotension and electrolytes disequilibrium • Require vascular access • Technically expertise required • Clearance rebound
**PIRRT/SLED**	Diffusion	6–12	• More rapid solutes removal than CRRT, but slower than IHD • More hemodynamically stable than IHD • Technically simpler than the techniques below • Relatively lower cost (e.g., less bags needed) • More restrictions on patients mobility than IHD but less than the techniques below	• Not recommended in critically ill hemodynamically unstable patients • Require vascular access and anticoagulant • Risks of hypotension and disequilibrium • Lower efficiency than other modalities
**CVVH**	Convection & ultrafiltration	24	• Convection allows highly efficient middle molecule and cytokine removal • Continuous removal of uremic toxins and fluid • Possible use in hemodynamic unstable patients	• Technically complex (e.g., complex circuit and high cost) • Require vascular access and anticoagulant (systemic or regional) • Need patient immobility • Prolonged exposure to membranes • Less efficient for small molecule removal than IHD
**CVVHD**	Diffusion & ultrafiltration	24	• Continuous removal of uremic toxins and fluid • Possible use in hemodynamic unstable patients • Better removal of small molecules than CVVH	• Technically complex (e.g., complex circuit and high cost) • Require vascular access and anticoagulant (systemic or regional) • Need patient immobility • Prolonged exposure to membranes • Less efficient for small molecule removal than IHD
**CVVHDF**	Diffusion, convection & ultrafiltration	24	• Convection allows highly efficient middle molecule and cytokine removal • Continuous removal of uremic toxins and fluid • Possible use in hemodynamic unstable patients • Better removal of small molecules than CVVH	• Technically most complex CRRT modality (e.g., complex circuit with frequent bag changes and high cost) • Require vascular access and anticoagulant (systemic or regional) • Need patient immobility • Prolonged exposure to membranes • Less efficient for small molecule removal than IHD

*PD *peritoneal dialysis, *IHD* intermittent hemodialysis, *PIRRT* prolonged intermittent renal replacement therapy, *SLED* slow low efficiency dialysis, *CVVH* continuous venovenous hemofiltration, *CVVHD* continuous venovenous hemodialysis, *CVVHDF* continuous venovenous hemodiafiltration

**Table 3:** Comparison between peritoneal dialysis and extracorporeal therapies main characteristics

**Characteristic**	**PD**	**IHD**	**CRRT**
**Duration**	Continuous for 24 h	Intermittent (4–6 h)	Continuous for 24 h
**Technical difficulties**	+	+ +	+ + +
**Influence on hemodynamics**	+	+ + +	+
**Control of fluid removal**	±	+ +	+ + +
**Possible catheter issues**	Obstruction, leak, peritonitis	Hemorrhage, thrombosis, dislocation	Hemorrhage, thrombosis, dislocation
**Availability**	+ + +	+ +	+
**Anticoagulation**	Not necessary	Variably needed	Needed
**Daily solute removal**	+	+ + +	+ + +
**Rapidity of solute clearance**	+	+ + +	+ +
**Easy to use in neonates**	+ + +	-	+

**Fig. 1:**
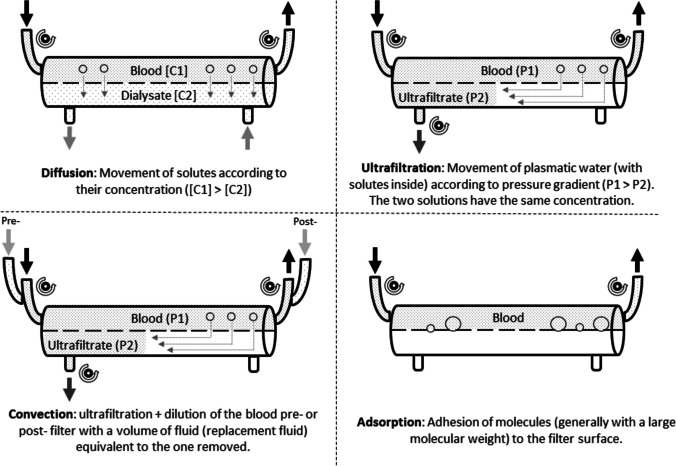
Physical principles of convection and diffusion

**Fig. 2:**
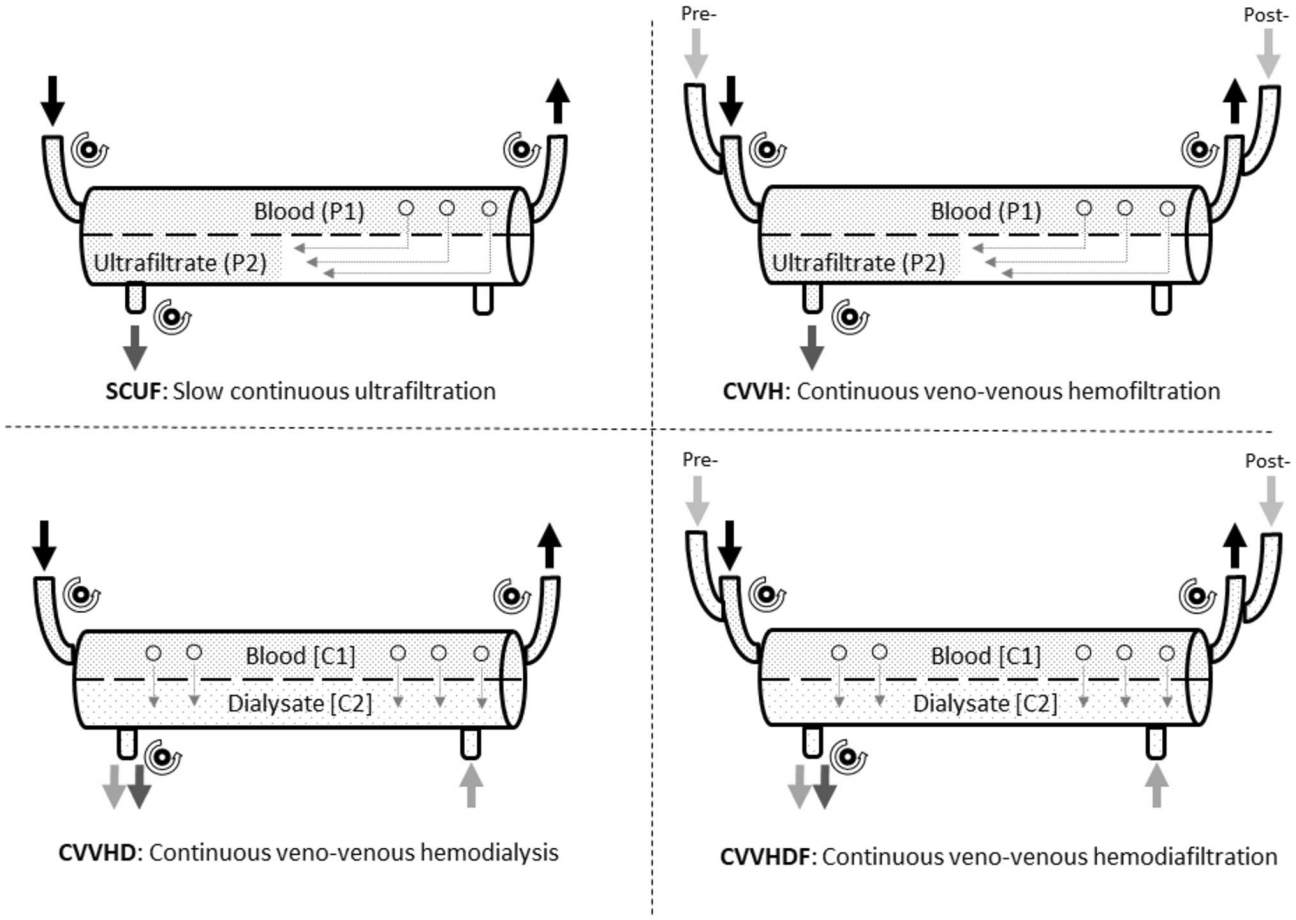
Schematic diagrams of modalities of CRRT

Regarding CRRT, a variety of modalities which differ in their mode of solute clearance may be used [8, 9, 58]. Figure 1 demonstrates the physical principles of diffusion and convection, while Fig. 2 illustrates the different modalities of CRRT. Continuous venovenous hemofiltration (CVVH) relies on the physical principle of convection, where ultrafiltration fluid is eliminated due to a hydrostatic gradient along the semi-permeable membrane and together with the fluid solutes is cleared, a mechanism called “solvent drag.” Convective techniques may provide enhanced elimination of middle molecular weight solutes like inflammatory mediators which might be beneficial in critically ill patients. As high ultrafiltrate rates are necessary to achieve sufficient solute clearance, a replacement fluid is administered pre- or postfilter (pre-dilution or post-dilution). On the other hand, continuous venovenous hemodialysis (CVVHD) relies on the principle of diffusion, where a dialysis fluid runs through the filter and molecules diffuse from blood to dialysate along a concentration gradient. Diffusive modalities allow for very effective clearance of small molecular weight solutes. Ultrafiltration rates are relatively low compared with convective modalities, which allows for fluid elimination without the need for replacement fluids. Continuous venovenous hemodiafiltration (CVVHDF) combines convection and diffusion to effectively clear both small and middle weight molecules and eliminate fluid. This method requires the administration of both a dialysis and a replacement fluid. Here again, the choice of the modality is dependent on the patient’s condition but also on the institutional preference and resources. Although CVVHDF seems the most effective choice as it combines filtration and dialysis and provides the broadest therapeutic options, it is also the most complex and resource-intense method as it requires frequent flow adjustments, monitoring of electrolytes, and fluid bag changes. Rarely performed is slow continuous ultrafiltration (SCUF), a method based on convection and used primarily for volume management without administration of a replacement fluid. It is used in a few clinical scenarios, for example, in a patient on ECMO to prevent or treat FO [8, 9]. The combined use of ECMO and CRRT is possible in different configurations. CRRT can be performed using a separate dialysis catheter or the CRRT device can be integrated into to the ECMO circuit by connecting the access and return line of CRRT before and after the oxygenator [59].

The CRRT dose corresponds to the effluent flow rate, which consists of the sum of dialysate and total ultrafiltrate flow. KDIGO practical guidelines recommend a delivered dose of 20–25 ml/kg/h [11]. Given the discrepancy between prescribed and delivered dose, a prescribed dose of 30–35 ml/kg/h is considered standard volume CRRT dose. High-volume CRRT (up to 80 ml/kg/h) may be necessary to increase ammonia clearance in newborns with inborn errors of metabolism or children with acute liver failure [60, 61]. Moreover, high volume CRRT has been shown to reduce vasopressor requirements and provide hemodynamic stability by removing inflammatory mediators in sepsis [62]. However, several large adult RCT’s have failed to demonstrate a difference in survival rates in patients treated with high volume compared to standard volume CRRT [63].

### Method of anticoagulation

The efficacy of CRRT is directly related to the longevity of the circuit as clotting of the circuit increases downtime, leads to blood loss of the patient, and may cause hemodynamic instability during de- and reconnection and increased costs [47, 64, 65]. To increase CL, anticoagulation of the extracorporeal circuit is necessary. Table 4 shows the most frequently used anticoagulation methods in pediatric CRRT and their advantages and disadvantages. Unlike adults, in children, due to small catheters and low blood flow rates, a strategy with no anticoagulation will result in short CL and thus ineffective CRRT [66]. The recent survey of ESPNIC Critical Care Nephrology Section demonstrated that the most frequently used anticoagulation methods in Europe are heparin (41% of responders) and regional citrate anticoagulation (RCA, 35%) [17]. While heparin is widely available, cheap, and easy to administer, it leads to systemic anticoagulation of the patient and thus may lead to bleeding complications. In children, heparin is mainly administered as unfractionated heparin (UFH) and rarely, unlike adults, as low molecular weight heparin (LMWH). In contrast, RCA is a strictly extracorporeal anticoagulation method and, in a recent review of all available pediatric studies, seems to reduce clotting events and prolong CL [67–70]. In adults, RCA has been shown to prolong CL in several prospective RCTs, and therefore, KDIGO recommends RCA as first-line anticoagulation method in patients who do not have a contraindication for citrate [71, 72]. However, RCA is a complex method and requires strict protocols, well-trained personnel, and frequent monitoring [73]. Whenever both heparin and RCA are contra-indicated or not available, prostacyclin can be used as alternative. Although not frequently used and therefore with limited experience, it seems a promising agent as it is easy to administer as continuous infusion into the circuit, does not require monitoring, and has a positive safety profile, with systemic hypotension being the most serious adverse event [74]. Deep et al. reported on their positive results using prostacyclin as anticoagulation method in children with acute liver failure [75]. More recently, a synthetic serine protease inhibitor, nafamostat, is being used as an alternative anticoagulant method with promising results among children receiving CRRT [76, 77].

**Table 4:** Most frequently anticoagulation methods used in pediatric CRRT and their advantages and disadvantages

**Method**	**Dosing (D) and monitoring (M)**	**Advantage**	**Disadvantage**
**Unfractionated heparin**	D: 10–20 IU/kg/h	• Easily reversible with protamine • Low costs and widely available • wide experience as anticoagulant	• Risk of patients bleeding • Patients possibly developing heparin induced thrombocytopenia (HIT) • Unpredictable and complex pharmacokinetics resulting in dosing variability
	M: aPTT 45–60 s or 1.5–2 × NR; ACT 180–200 s		
**Low Molecular Weight Heparin**	D: Enoxaparin LD 0.15 mg/kg, MD 0.05 mg/kg/h	• Less risks for HIT • Pharmacokinetics more predictable than unfractionated heparin	• Higher costs than unfractionated heparin • Less effective reversal with protamine
	M: Anti-Xa level (0.3–0.7 UI/mL)		
**Regional citrate anticoagulation**	D: starting dose 3 mmol/L^a^	• Anticoagulation only of the extracorporeal circuit • Lower risks of bleeding • Longer filter life than heparin	• Need for training and strict protocols • Higher risks of citrate complications (electrolytes imbalance, citrate accumulation/toxicity) • Need for high dialytic dose (high volume of pre-filter fluid) • May need caution in patients with severe liver failure and lactic acidosis
	M: extracorporeal iCa 0.25–0.35 mmol/L; intracorporeal iCa 1.1–1.3 mmol/L		
**Regional heparin and protamine**	D: infuse 1 mg protamine post-filter for 100 IU Heparin	• Anticoagulation only of the extracorporeal circuit • Lower risks of bleeding	• Complex metabolism may lead to prolonged anticoagulation • Requires measurement of both circuit and patient APTT • Technically challenging (difficulty in estimating the amount of protamine required to antagonize post-filter heparin) • Possible side effects: hypotension, anaphylaxis, cardiac depression, leukopenia, and thrombocytopenia
	M: circuit aPTT 45–60 s or 1.5–2 × NR; ACT 180–200		
**Prostacyclin infusion**	D: 2–8 ng/kg/min	• No need for anticoagulation parameter monitoring since inhibits platelets aggregation • Easy to perform	• Possible hemodynamic impact, dose dependent (vasodilation, systemic hypotension, possible reflex tachycardia) • Possible raised intracranial pressure
	M: no monitoring tests		
**Serine protease inhibitors—nafamostat mesilate, aprotinin**	D: Depending on drug	• Lower costs than regional citrate anticoagulation • Alternative to regional citrate anticoagulation if risk of citrate accumulation	• Only few studies available in pediatrics • Need for clotting parameter monitoring
	M: aPTT 45–60 s or 1.5–2 × NR; ACT 180–200 s		
**Direct thrombin inhibitors—argatroban, bivalirudin**	D: Depending on drug	• Lower bleeding risk than unfractionated heparin in other context (e.g., ECMO) • Shorter half-life than heparin (bivalirudin the shortest) • Possible use in patients with HIT	• Only few studies available in pediatrics, evidences from adults • Non-reversible agents available
	M: aPTT 45–60 s or 1.5–2 × NR; ACT 180–200 s		

*LD* loading dose, *MD* maintenance dose, *NR* normal range

^a^Citrate flow rate depends on the type of citrate solution used

### Dedicated neonatal and pediatric machines

Most commercially available CRRT machines are not designed and not licensed for smaller children. However, it should be mentioned that due to technological refinements in modern machines resulting in circuits with smaller extracorporeal volumes and accurate ultrafiltration rates, a high safety level has been achieved [15, 78]. However, some concerns remain in small children with a body weight of less than 8 kg. Recently, a dedicated neonatal of infant dialysis machine called CARPEDIEM^®^ has become available and, according to the recent ESPNIC survey, is used in Europe by up to 15% centers [17, 79–81]. This machine allows for safe administration of CRRT in newborns due to its accuracy and very small extracorporeal volumes: On the other hand, the diminutive design limits treatment options and does not allow RCA [79, 80]. Moreover, a second machine is needed for larger children and thus the team needs training on two different machines. Therefore, the use of dedicated neonatal machines might be limited to centers with a large neonatal or infant population, for example, centers with a large congenital cardiac surgery program [81].

### Liberation from CRRT

Discontinuation of CRRT, as per the KDIGO guidelines, should be considered “when CRRT is no longer required either because intrinsic kidney function has recovered to the point that is adequate to meet patient needs, or because CRRT is no longer consistent with the goal of care” [11]. These guidelines also state that the use of diuretics is not recommended to enhance kidney function recovery, or to reduce the duration of CRRT. The clinical indicators for discontinuation of CRRT include increased urinary output, no more fluid overload, and the patient is no longer receiving vasoactive medications. Clinician should consider “filter holiday” if spontaneous urine output is > 0.5 mL/kg/h and fluid status, acid–base status, and electrolytes are controlled. The ESPNIC survey confirms that among the abovementioned, the increase of the native urine output and the resolution of FO were the 2 factors most often associated with the decision to perform a trial of liberation from CKRT [17].

## Outcomes

### In hospital outcomes

Several studies have reported on the outcomes of critically ill children requiring CRRT over the last 20 years [6, 12, 24–26, 29, 30, 82, 83]. Overall reported PICU mortality in these studies was high and ranged from 35 to 64%, and thus rivals mortality in adults. However, most of these studies were single-center studies and the patient population differed significantly between studies. Two larger multicenter studies from the North American prospective pediatric continuous renal replacement registry (ppCRRT) found mortality rates of 42 and 43%, respectively [25, 82]. In more recent studies, however, mortality seems to have decreased slightly [29, 84]. This effect may be caused by the overall increased use of CRRT among less sick children and/or earlier initiation of CRRT due to better recognition of AKI in critically ill children. Critically ill children requiring CRRT are a heterogeneous group with different diagnosis, and several pediatric studies have shown that outcome is mainly related to the underlying disease, severity of illness, presence of MODS, and the degree of FO at CRRT initiation. Highest mortality rates have been described in children with onco-hematologic disease (50–80%), especially after stem cell transplantation, in patients with liver disease (50–69%), cardiac disease/surgery (35–62%), and sepsis (33–44%) [4, 24, 29, 30, 82, 85]. On the other hand, excellent outcomes with low mortality rates have been reported in children with metabolic disease (10–27%) and primary renal disease (6–34%). In addition to increased mortality, CRRT has been associated with increased length of mechanical ventilation and ICU stay [24, 29].

### Long-term outcomes

Historically, it was believed that patients who recovered kidney function after AKI had benign long-term outcomes. There is growing evidence demonstrating that these children are also at risk for adverse long-term outcomes such as CKD, proteinuria, hypertension, increased healthcare utilization, and mortality [86–89]. In a systematic review of pediatric AKI studies, the pooled long-term incidence of proteinuria was 13%, hypertension 7%, abnormal GFR (< 90 mL/min/1.73m^2^) 28%, and end-stage kidney disease 0.4% [87]. Together, these studies highlight the importance of kidney health surveillance after episodes of childhood AKI.

### Follow-up of AKI survivors

Current AKI follow-up care is inadequate due to low rates of AKI recognition in hospitalized children, suboptimal documentation of AKI events and follow-up recommendations in discharge summaries, lack of awareness of AKI and its consequences at both patient and provider level, lack of clear post-AKI follow-up care guidelines, and limited access to pediatric nephrology clinics.

In a population-based study in Ontario, Canada, from 1996 to 2017 involving ~ 1700 children who received dialysis for AKI, nephrology follow-up was suboptimal (19% by 1 year and 27% by 10 years) [88]. However, most dialysis-treated AKI survivors (97%) had at least one outpatient physician visit by 1 year. Similarly, < 25% of PICU patients with AKI were followed up by a pediatric nephrologist in a 5-year period after discharge but > 95% had an outpatient physician visit within 1 year [90]. These data suggest that general pediatricians and primary care providers should be targeted for knowledge translation strategies related to post-AKI follow-up care.

Strategies to improve post-AKI follow-up must be initiated at the time of hospitalization and should include increased recognition (e.g., electronic health record alerts and provider education), improving documentation of AKI episode in discharge summaries, effective communication to primary care providers regarding care plan, and education of patients and their families about the AKI event, potential long-term risks, and need for regular follow-up.

However, the optimal timing and content of post-AKI follow-up care remains unclear. KDIGO guidelines suggest evaluating “patients 3 months after AKI for resolution, new onset, or worsening of pre-existing CKD” (ungraded recommendation) [11]. Generally, children with severe AKI (stage 3 or receiving dialysis), prolonged AKI duration (≥ 7 days), and/or incomplete recovery should be re-assessed soon after the discharge and nephrologist referral should also be considered for these children. General pediatricians or primary care providers can follow-up those with less severe AKI.

## Conclusion

This review, from the Critical Care Nephrology section of the European Society of Paediatric and Neonatal Intensive Care (ESPNIC), provides an overview of current recommendations regarding key aspects of CRRT delivery which might be of interest for general pediatricians. Additionally, we intended to stress the importance of adequate follow-up of PICU patients with AKI and CRRT as recent findings demonstrate that these children are at increased risk for adverse long-term outcomes.
